# Hospital Residents and Economy

**Published:** 1907-05-04

**Authors:** 


### Hospital Residents and Economy.

Mr. J. Langford Moore writes from St. Bartholomew's Hospital, April 24:

In your instructive article on the above in the current issue of your journal, with reference to economy in drugs it is stated that "no teacher of materia medica or therapeutics, in his lectures on these subjects, indicates to his pupils the fact that some drugs are more costly than others, and that some are very costly indeed." Now, in most hospitals with medical schools attached, the teacher in materia medica and pharmacy is usually the pharmacist to the hospital, and at some period or other during his curriculum practically every resident medical officer, house physician, or house surgeon must have attended his classes. The keen pharmacist, a person obviously well acquainted with existing drug prices, having an eye to the future expenses of his department, avails himself of the opportunity presented to inculcate the habit of economic care in the prescriptions of his future staff.

In the hospital it is my privilege to serve, the rule is adopted that when an expensive drug is first ordered the prescriber is seen, the cost intimated, and a list supplied of the relative prices of drugs having similar therapeutic action; in this way a considerable saving may be effected. Frequently the same synthetic article may appear in drug catalogues under half a dozen different names, given to it by various makers, and with a like variance in price; a good example is afforded by the numerous organic urinary antiseptics in use at the present time.

In an experience of hospital work extending over some years, I have found that by means such as these the hearty co-operation of the staff is enlisted, extravagance through lack of knowledge avoided, and the medical man prepared for the future when his drug bill may be a matter for careful consideration.

May I be permitted to add that the various articles on hospital administration appearing from time to time in your well-informed journal cannot but be of the greatest possible use to all interested in hospital work, whether medical or lay?

Sheffield Union Hospital.—This matter is dealt with on page 114.

